# Real-world outcomes with avelumab + axitinib in patients with advanced renal cell carcinoma in Japan: subgroup analyses from the J-DART2 study by International Metastatic Renal Cell Carcinoma Database Consortium risk classification

**DOI:** 10.1007/s10147-024-02655-4

**Published:** 2025-02-11

**Authors:** Junya Furukawa, Taigo Kato, Toshinari Yamasaki, Keisuke Monji, Toshiaki Tanaka, Norihiko Tsuchiya, Tomoaki Miyagawa, Hiroshi Yaegashi, Tomoyasu Sano, Takashi Karashima, Kazutoshi Fujita, Jun-ichi Hori, Takayuki Ito, Masahiro Kajita, Yoshihiko Tomita, Nobuo Shinohara, Masatoshi Eto, Mototsugu Oya, Hirotsugu Uemura

**Affiliations:** 1 Department of Urology, Kobe University Graduate School of Medicine, 7-5-1 Kusunoki-Cho, Chuo-ku, Kobe, 650-0017 Japan; 2 Present Address: Department of Urology, Tokushima University, Graduate School of Biomedical Sciences, 3-18-15 Kuramoto-cho, Tokushima, 770-8503 Japan; 3 Department of Urology, Osaka University Graduate School of Medicine, 2-2 Yamadaoka, Suita, Osaka 565-0871 Japan; 4 Department of Urology, Kobe City Medical Center General Hospital, 2-1-1 Minatojima Minamimachi, Chuo-ku, Kobe, 650-0047 Japan; 5 Department of Urology, Graduate School of Medical Sciences, Kyushu University, 3-1-1 Maidashi, Higashi-ku, Fukuoka, 812-8582 Japan; 6 Department of Urology, Sapporo Medical University School of Medicine, South-1, West-16, Chuo-ku, Sapporo, Hokkaido 060-8543 Japan; 7 Department of Urology, Faculty of Medicine, Yamagata University, 2-2-2, Iida-Nishi, Yamagata 990-9585 Japan; 8 Department of Urology, Jichi Medical University Saitama Medical Center, 1-847, Amanuma-cho, Omiya-ku, Saitama-shi, Saitama, 330-8503 Japan; 9 Department of Integrative Cancer Therapy and Urology, Kanazawa University Graduate School of Medical Sciences, 13‐1 Takaramachi, Kanazawa City, Ishikawa 920-8641 Japan; 10 Department of Urology, Nagoya University Graduate School of Medicine, 65 Tsurumai-cho, Showa-ku, Nagoya, 466-8550 Japan; 11 Department of Urology, Kochi Medical School, Kohasu, Oku-cho, Nankoku, Kochi 783-8505 Japan; 12 Department of Urology, Kindai University Faculty of Medicine, 377-2 Ohno-Higashi, Osakasayama City, Osaka, 589-8511 Japan; 13 Department of Renal and Urologic Surgery, Asahikawa Medical University, 2-1-1-1 Midorigaoka Higashi, Asahikawa, Hokkaido 078-8510 Japan; 14 Medical Department, Merck Biopharma Co., Ltd., Tokyo, Japan, an affiliate of Merck KGaA, 1-8-1 Shimomeguro, Meguro-ku, Tokyo, 153-8926 Japan; 15 Departments of Urology and Molecular Oncology, Niigata University Graduate School of Medical and Dental Sciences, 1-757 Asahimachidori Chuo-ku, Niigata, 951-8510 Japan; 16 Department of Renal and Genitourinary Surgery, Graduate School of Medicine, Hokkaido University, Kita15, Nishi7, Kita-ku, Sapporo, Hokkaido 060-8638 Japan; 17 Department of Urology, Keio University School of Medicine, 35 Shinanomachi, Shinjuku-ku, Tokyo, 160-8582 Japan

**Keywords:** Renal cell carcinoma, Avelumab, Axitinib, IMDC risk classification, Real world, Retrospective

## Abstract

**Background:**

Avelumab + axitinib was approved for the treatment of advanced renal cell carcinoma (aRCC) in Japan in December 2019. We report long-term real-world subgroup analyses with first-line avelumab + axitinib in patients with aRCC by International Metastatic RCC Database Consortium (IMDC) risk classification from the J-DART2 study in Japan.

**Methods:**

J-DART2 was a multicenter, noninterventional, retrospective study examining characteristics, treatment patterns, and outcomes in patients with aRCC who started first-line avelumab + axitinib in Japan between December 2019 and October 2022.

**Results:**

Data from 150 patients across 19 sites were analyzed. IMDC risk was favorable in 39 patients (26.0%), intermediate (1 risk factor) in 46 (30.7%), intermediate (2 risk factors) in 36 (24.0%), and poor in 29 (19.3%). Baseline characteristics were generally consistent across IMDC risk subgroups. In subgroups with favorable, intermediate (1 risk factor), intermediate (2 risk factors), and poor risk, median progression-free survival was 31.0, 15.3, 16.4, and 8.1 months; median overall survival (OS) was not reached, but 24-month OS rates were 95.2%, 91.3%, 85.3%, and 57.6%, respectively. Objective response rates were 54.5%, 56.8%, 47.1%, and 54.2%, respectively. High-dose corticosteroid treatment for immune-related adverse events was administered in 5.1%, 8.7%, 8.3%, and 6.9% of patients, respectively.

**Conclusion:**

Subgroup analyses from J-DART2 confirm the long-term real-world effectiveness of first-line avelumab + axitinib across IMDC risk groups in patients with aRCC in Japan. Our findings were consistent with previous analyses by IMDC risk and support the favorable benefit-risk profile of avelumab + axitinib in clinical practice in Japan.

**Supplementary Information:**

The online version contains supplementary material available at 10.1007/s10147-024-02655-4.

## Introduction

Combination treatment involving an immune checkpoint inhibitor (ICI) is the standard-of-care first-line (1L) treatment for patients with advanced renal cell carcinoma (aRCC) [1–3]. The International Metastatic RCC Database Consortium (IMDC) risk classification is a commonly used prognostic model that helps guide treatment strategies for patients with aRCC [4, 5]. The IMDC risk classification uses 6 factors to categorize patients as having favorable (0 risk factors), intermediate (1 or 2 risk factors), or poor (≥ 3 risk factors) risk disease. These factors include the time interval from diagnosis to systemic treatment, Karnofsky performance status, hemoglobin level, platelet count, neutrophil count, and serum calcium concentration. Current guidelines for the 1L treatment of aRCC recommend several options of ICI + tyrosine kinase inhibitor (TKI) combinations across all IMDC risk groups [1–3]; thus, there is a need to determine the optimal ICI + TKI combination for each patient. Analyses of outcomes according to the number of IMDC risk factors may help guide more individualized strategies for patients in each risk group.

Combination treatment with avelumab, an ICI that inhibits programmed death ligand 1, and axitinib, a multitargeted TKI that inhibits vascular endothelial growth factor receptors, is approved as a 1L treatment for patients with aRCC in various countries worldwide, including Japan [6–10]. This approval was based on results from the phase 3 JAVELIN Renal 101 trial (NCT02684006), which demonstrated significantly longer median progression-free survival (PFS) and a higher objective response rate (ORR) with avelumab + axitinib vs sunitinib, the prior standard of care in aRCC; median overall survival (OS) favored avelumab + axitinib vs sunitinib, but differences did not reach statistical significance [11–14]. In post hoc analyses from JAVELIN Renal 101, hazard ratios (HRs) for PFS and OS favored avelumab + axitinib vs sunitinib in patients with IMDC favorable-, intermediate-, or poor-risk disease, and a higher proportion of patients in the combination arm had an objective response across all risk groups [13]; similar results were observed in post hoc analyses by the number of IMDC risk factors (1, 2, 3, or ≥ 4) [15].

The JAVELIN Renal 101 study included 67 patients who were enrolled in Japan, and subgroup analyses in this population showed improved PFS and ORR with avelumab + axitinib vs sunitinib [16]. The data from JAVELIN Renal 101 led to the approval of avelumab + axitinib in Japan in December 2019 for patients with curatively unresectable or metastatic RCC [10]. Consequently, the Japanese Urological Association clinical practice guidelines for RCC recommend avelumab + axitinib for the 1L treatment of patients with clear cell RCC [2]. However, the number of patients enrolled in the avelumab + axitinib arm of JAVELIN Renal 101 in Japan was limited, and real-world data are needed to assess the effectiveness of 1L avelumab + axitinib in patients with aRCC receiving routine clinical care in Japan, including outcomes by IMDC risk classification.

Thus far, real-world postmarketing surveillance in Japan confirmed the safety and effectiveness of 1L avelumab + axitinib in patients with aRCC (observation period, ≤ 1 year) [17]; however, data on patient outcomes according to IMDC risk classification are needed. The real-world J-DART study (NCT05012865) showed clinically meaningful benefits in patients (N = 48) with aRCC treated with 1L avelumab + axitinib 1 year after its approval in Japan, but the study was limited by the small patient number [18]. In the larger observational J-DART2 study (NCT05650164; N = 150), 1L avelumab + axitinib was associated with clinically meaningful benefits in the overall population and across age groups [19]. Here, we report long-term (observation period, ≥ 2 years) outcomes by IMDC risk classification in patients with aRCC treated with 1L avelumab + axitinib in Japan from the J-DART2 study, including patients with favorable, intermediate (1 or 2 risk factors), or poor risk.

## Patients and methods

### Study design

The study design of J-DART2 has been described previously [19]. Briefly, J-DART2 (NCT05650164) was a multicenter, observational, retrospective study performed at 19 sites in Japan. Clinical data were collected from patients aged ≥ 18 years with curatively unresectable locally advanced or metastatic RCC (based on the General Rule for Clinical and Pathological Studies on Renal Cell Carcinoma [5th edition]) who started treatment with 1L avelumab + axitinib between 20 December 2019 (approval date) and 17 October 2022. The follow-up period was from the date of the first prescription until 31 October 2022. Evidence of signed or oral consent was obtained for surviving patients, and evidence of consent from a family member was obtained for deceased patients. The study excluded patients participating in a prospective interventional clinical trial during the follow-up period. Data were collected from patient medical records within the follow-up period. All decisions regarding the treatment and clinical management of patients were made by the investigator as part of standard clinical care in a real-world setting and irrespective of the patient’s participation in the study. Ethical review boards from all study sites approved the study protocol and related documentation. The study conduct complied with the Declaration of Helsinki and applicable local laws in Japan.

### Objectives and assessments

The primary objective was to describe the demographic and clinical characteristics of patients with aRCC treated with 1L avelumab + axitinib in clinical practice in Japan. The secondary objective was to determine real-world treatment outcomes as measured by endpoints that included ORR and PFS per investigator assessment, OS, treatment exposure, use of corticosteroid treatments for immune-related adverse events (irAEs), and subsequent treatment patterns. Results are reported in subgroups of patients defined by IMDC risk classification, consisting of favorable, intermediate (1 risk factor), intermediate (2 risk factors), or poor risk.

### Statistical analysis

The full analysis population included all enrolled patients at each site during the study period. Effectiveness was assessed in all patients from the full analysis population whose index date was prior to 30 April 2022 to ensure a 6-month follow-up period. Continuous variables were summarized using descriptive statistics. Qualitative variables were summarized as frequencies and percentages. Time-to-event endpoints (PFS and OS) were estimated using the Kaplan–Meier method, and corresponding CIs were calculated using the Brookmeyer-Crowley method. Median duration of avelumab + axitinib follow-up was determined by the reverse Kaplan-Meler method. Statistical analyses were performed using SAS 9.4 (SAS Institute, Inc).

An exploratory multivariable logistic regression analysis was performed using Cox regression to examine the association of baseline C-reactive protein (CRP) levels, estimated glomerular filtration rate (eGFR), and IMDC risk with OS and PFS. Patients were categorized as having CRP levels < 10 mg/L or ≥ 10 mg/L. The cutoff value for the CRP level was set at 10 mg/L, which is considered high based on a standard CRP test and was used in previously published studies [20, 21]. Patients were categorized as having eGFR levels < 30 mL/min, 30–60 mL/min, or ≥ 60 mL/min.

## Results

### Patients and treatment

At data cutoff (31 October 2022), 150 patients from 19 sites were included in the study. Median duration of follow-up was 18.7 months (95% CI, 16.3–20.6). IMDC risk classification was favorable in 39 patients (26.0%), intermediate (1 risk factor) in 46 (30.7%), intermediate (2 risk factors) in 36 (24.0%), and poor in 29 (19.3%). The distribution of risk factors in patients with intermediate (1 or 2 risk factors) or poor risk is shown in the Supplementary Table. In patients with intermediate risk (1 risk factor), 24 (52.2%) had < 1 year from time of diagnosis to systemic therapy, 17 (37.0%) had hemoglobin level < lower limit of normal, and 5 (10.9%) had neutrophil count > upper limit of normal. In patients with intermediate risk (2 risk factors), 23 (63.9%) had < 1 year from time of diagnosis to systemic therapy and hemoglobin level < lower limit of normal, and 6 (16.7%) had < 1 year from time of diagnosis to systemic therapy plus a risk factor other than hemoglobin level < lower limit of normal. In patients with poor risk, 20 (69.0%) had < 1 year from time of diagnosis to systemic therapy, hemoglobin level < lower limit of normal, plus another risk factor.

**Fig. 1:**
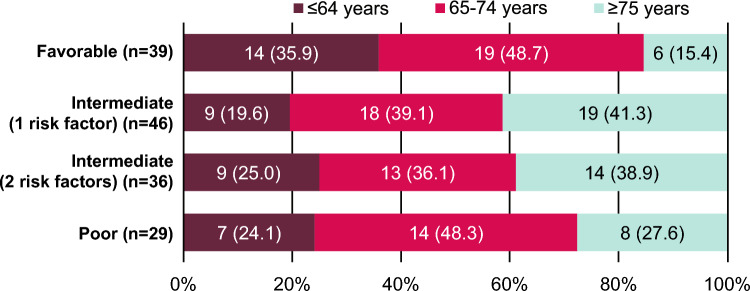
Age distribution in subgroups defined by IMDC risk classification. *IMDC* International Metastatic renal cell carcinoma Database Consortium

Baseline characteristics were generally consistent across IMDC risk subgroups (Table 1, Fig. 1). In patients with favorable risk, 14 (35.9%) were aged ≤ 64 years, 19 (48.7%) were aged 65–74 years, and 6 (15.4%) were aged ≥ 75 years. In patients with intermediate risk (1 risk factor), 9 (19.6%) were aged ≤ 64 years, 18 (39.1%) were aged 65–74 years, and 19 (41.3%) were aged ≥ 75 years. In patients with intermediate risk (2 risk factors), 9 (25.0%) were aged ≤ 64 years, 13 (36.1%) were aged 65–74 years, and 14 (38.9%) were aged ≥ 75 years. In patients with poor risk, 7 (24.1%) were aged ≤ 64 years, 14 (48.3%) were aged 65–74 years, and 8 (27.6%) were aged ≥ 75 years.

**Table 1:** Baseline characteristics in subgroups defined by IMDC risk classification

	Favorable (n = 39)	Intermediate (1 risk factor) (n = 46)	Intermediate (2 risk factors) (n = 36)	Poor (n = 29)
Sex, n (%)				
Male	26 (66.7)	33 (71.7)	28 (77.8)	23 (79.3)
Female	13 (33.3)	13 (28.3)	8 (22.2)	6 (20.7)
Age				
Median (range), years	68 (37–84)	73 (49–86)	71 (42–87)	70 (33–82)
≤ 64 years, n (%)	14 (35.9)	9 (19.6)	9 (25.0)	7 (24.1)
65–74 years, n (%)	19 (48.7)	18 (39.1)	13 (36.1)	14 (48.3)
≥ 75 years, n (%)	6 (15.4)	19 (41.3)	14 (38.9)	8 (27.6)
BMI, n (%)				
< 25 kg/m^2^	25 (64.1)	33 (71.7)	25 (69.4)	27 (93.1)
≥ 25 kg/m^2^	13 (33.3)	13 (28.3)	11 (30.6)	2 (6.9)
ECOG PS, n (%)				
0	35 (89.7)	38 (82.6)	25 (69.4)	18 (62.1)
1	4 (10.3)	8 (17.4)	8 (22.2)	3 (10.3)
≥ 2	0	0	2 (5.6)	8 (27.6)
CRP, n (%)				
< 10 mg/L	37 (94.9)	40 (87.0)	19 (52.8)	8 (27.6)
≥ 10 mg/L	2 (5.1)	4 (8.7)	16 (44.4)	21 (72.4)
eGFR, n (%)				
< 30 mL/min	2 (5.1)	6 (13.0)	4 (11.1)	4 (13.8)
30–60 mL/min	27 (69.2)	32 (69.6)	21 (58.3)	13 (44.8)
≥ 60 mL/min	10 (25.6)	7 (15.2)	11 (30.6)	12 (41.4)
Pathological classification, n (%)				
Clear cell	38 (97.4)	41 (89.1)	32 (88.9)	23 (79.3)
Non-clear cell	0	5 (10.9)	0	1 (3.4)
Unknown	1 (2.6)	0	4 (11.1)	5 (17.2)
Sarcomatoid, n (%)	2 (5.1)	4 (8.7)	3 (8.3)	1 (3.4)
Metastatic lesion, n (%)				
0	3 (7.7)	0	3 (8.3)	3 (10.3)
1	18 (46.2)	29 (63.0)	17 (47.2)	9 (31.0)
≥ 2	18 (46.2)	17 (37.0)	16 (44.4)	17 (58.6)
Nephrectomy, n (%)	38 (97.4)	41 (89.1)	23 (63.9)	12 (41.4)
Comorbidities, n (%)	22 (56.4)	32 (69.6)	27 (75.0)	20 (69.0)
IMDC risk factors, n (%)				
< 1 year to therapy	0	24 (52.2)	29 (80.6)	23 (79.3)
Hemoglobin level < normal	0	17 (37.0)	29 (80.6)	26 (89.7)
Karnofsky PS < 80%	0	0	3 (8.3)	8 (27.6)
Calcium level > normal	0	0	3 (8.3)	14 (48.3)
Neutrophil level > normal	0	5 (10.9)	4 (11.1)	13 (44.8)
Platelet count > normal	0	0	4 (11.1)	14 (48.3)

Patients with unknown classification are not included in relevant rows

*BMI* body mass index, *CRP* C-reactive protein, *ECOG* Eastern Cooperative Oncology Group, *eGFR* estimated glomerular filtration rate, *IMDC* International Metastatic renal cell carcinoma Database Consortium, *PS* performance status

The poor-risk subgroup had a higher proportion of patients with Eastern Cooperative Oncology Group performance status of ≥ 2 vs the subgroups with favorable, intermediate (1 risk factor), or intermediate (2 risk factors) risk (27.6% vs 0%, 0%, or 5.6%, respectively), a higher proportion of patients with CRP level of ≥ 10 mg/L (72.4% vs 5.1%, 8.7%, or 44.4%, respectively), a higher proportion of patients with ≥ 2 metastatic lesions (58.6% vs 46.2%, 37.0%, or 44.4%, respectively), and a lower proportion of patients with prior nephrectomy (41.4% vs 97.4%, 89.1%, or 63.9%) (Table 1).

Median duration of avelumab + axitinib treatment in subgroups with favorable, intermediate (1 risk factor), intermediate (2 risk factors), and poor risk was 12.6 months (IQR, 6.1–20.1), 12.0 months (IQR, 8.1–17.0), 10.5 months (IQR, 7.3–17.8), and 8.0 months (IQR, 2.6–14.3), respectively (Table 2). A similar trend was observed for median duration of treatment with avelumab alone or axitinib alone. In patients in the subgroups with favorable, intermediate (1 risk factor), intermediate (2 risk factors), and poor risk, median relative dose intensity for avelumab was 100%, 100%, 95%, and 100%, respectively, and median relative dose intensity for axitinib was 80%, 70%, 70%, and 90%.

**Table 2:** Treatment exposure for avelumab and axitinib in subgroups defined by IMDC risk classification

	Favorable (n = 39)	Intermediate (1 risk factor) (n = 46)	Intermediate (2 risk factors) (n = 36)	Poor (n = 29)
Duration of treatment, median (IQR), months				
Avelumab + axitinib	12.6 (6.1-20.1)	12.0 (8.1-17.0)	10.5 (7.3-17.8)	8.0 (2.6-14.3)
Avelumab	11.7 (5.1-19.5)	11.2 (7.9-15.7)	9.9 (6.7-16.6)	7.6 (2.6-10.5)
Axitinib	11.5 (5.7-19.0)	10.8 (6.9-15.7)	9.3 (4.1-16.7)	6.9 (2.3-10.5)
Avelumab dose, median (IQR)				
DI, mg/kg/administration	10.0 (9.4-10.0)	10.0 (8.1-10.0)	9.5 (8.0-10.0)	10.0 (8.6-10.0)
RDI, %	100.0 (94.0-100.0)	100.0 (80.8-100.0)	95.0 (80.0-100.0)	100.0 (86.0-100.0)
Axitinib dose, median (IQR)				
DI, mg/administration	6.1 (4.3-8.9)	5.2 (3.9-6.8)	4.8 (3.4-7.0)	5.3 (4.0-7.2)
RDI, %	80.0 (60.0-100.0)	70.0 (50.0-97.5)	70.0 (60.0-100.0)	90.0 (60.0-100.0)

*DI* dose intensity, *IMDC* International Metastatic renal cell carcinoma Database Consortium, *RDI* relative dose intensity

### Clinical outcomes

**Fig. 2:**
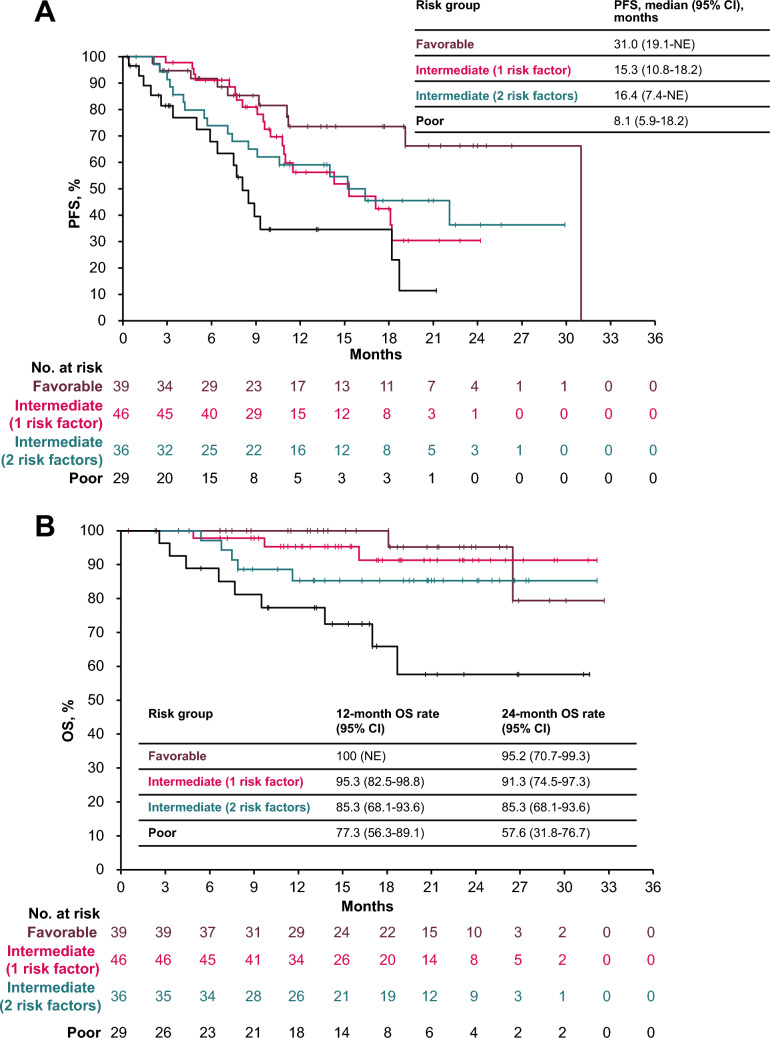
Real-word PFS (**a**) and OS (**b**) in subgroups defined by IMDC risk classification. *IMDC* International Metastatic renal cell carcinoma Database Consortium, *NE* not evaluable, *OS* overall survival, *PFS* progression-free survival

Median PFS in subgroups with favorable, intermediate (1 risk factor), intermediate (2 risk factors), and poor risk was 31.0 months (95% CI, 19.1-not estimable), 15.3 months (95% CI, 10.8–18.2), 16.4 months (95% CI, 7.4-not estimable), and 8.1 months (95% CI, 5.9–18.2), respectively (Fig. 2A). In a multivariable analysis, the HR for PFS in the poor- vs favorable-risk subgroup was 3.825 (95% CI, 1.533–9.544; p = 0.0040) (Table 3).

**Table 3:** Multivariable analyses for PFS

Baseline characteristics	Events, n (%)	Median (95% CI), months	HR (95% CI)	p value
CRP				
< 10 mg/L (n = 104)	41 (39.4)	22.1 (11.5-NE)	Reference	
≥ 10 mg/L (n = 43)	25 (58.1)	9.1 (7.5-15.2)	1.397 (0.760-2.566)	0.2813
Unknown (n = 3)	0	NE (NE-NE)	NE (NE-NE)	NE
eGFR				
≥ 60 mL/min (n = 40)	18 (45.0)	15.3 (14.0-NE)	Reference	
≥ 30 and < 60 mL/min (n = 93)	38 (40.9)	18.7 (10.8-NE)	0.976 (0.549-1.735)	0.9340
< 30 mL/min (n = 16)	10 (62.5)	11.0 (4.7-18.1)	1.772 (0.784-4.002)	0.1689
Unknown (n = 1)	0	NE (NE-NE)	NE (NE-NE)	NE
IMDC risk classification				
Favorable (n = 39)	10 (25.6)	31.0 (19.1-NE)	Reference	
Intermediate (1 risk factor) (n = 46)	21 (45.7)	15.3 (10.8-18.2)	1.992 (0.895-4.438)	0.0916
Intermediate (2 risk factors) (n = 36)	18 (50.0)	16.4 (7.4-NE)	1.929 (0.829-4.486)	0.1271
Poor (n = 29)	17 (58.6)	8.1 (5.9-18.2)	3.825 (1.533-9.544)	0.0040

*CRP* C-reactive protein, *eGFR* estimated glomerular filtration rate, *HR* hazard ratio, *IMDC* International Metastatic renal cell carcinoma Database Consortium, *NE* not estimable, *PFS* progression-free survival

Median OS was not reached in any subgroup; however, 12-month OS rates in subgroups with favorable, intermediate (1 risk factor), intermediate (2 risk factors), and poor risk were 100%, 95.3%, 85.3%, and 77.3%, respectively, and 24-month OS rates were 95.2%, 91.3%, 85.3%, and 57.6%, respectively (Fig. 2B). In a multivariable analysis, the HR for OS in the poor- vs favorable-risk subgroup was 6.323 (95% CI, 1.064–37.563; p = 0.0425), and the HR for OS in patients with estimated glomerular filtration rate of < 30 vs > 60 mL/min was 9.940 (95% CI, 2.148–46.004; p = 0.0033) (Table 4).

**Table 4:** Multivariable analyses for OS

Baseline characteristics	Events, n (%)	12-month OS rate (95% CI)	24-month OS rate (95% CI)	HR (95% CI)	p value
CRP					
< 10 mg/L (n = 104)	8 (7.7)	94.8 (87.9-97.8)	91.6 (82.8-96.0)	Reference	
≥ 10 mg/L (n = 43)	11 (25.6)	79.0 (62.2-88.9)	64.2 (42.4-79.5)	1.816 (0.593-5.563)	0.2962
Unknown (n = 3)	0	100.0 (100.0-100.0)	100.0 (100.0-100.0)	NE (NE-NE)	NE
eGFR					
≥ 60 mL/min (n = 40)	3 (7.5)	94.6 (80.0-98.6)	94.6 (80.0-98.6)	Reference	
≥ 30 and < 60 mL/min (n = 93)	11 (11.8)	92.0 (84.0-96.1)	84.8 (73.7-91.5)	2.431 (0.669-8.824)	0.1770
< 30 mL/min (n = 16)	5 (31.3)	71.8 (41.1-88.4)	61.5 (29.7-82.4)	9.940 (2.148-46.004)	0.0033
Unknown (n = 1)	0	100.0 (100.0-100.0)	NE (NE-NE)	NE (NE-NE)	NE
IMDC risk classification					
Favorable (n = 39)	2 (5.1)	100.0 (100.0-100.0)	95.2 (70.7-99.3)	Reference	
Intermediate (1 risk factor) (n = 46)	3 (6.5)	95.3 (82.5-98.8)	91.3 (74.5-97.3)	0.924 (0.149-5.717)	0.9326
Intermediate (2 risk factors) (n = 36)	5 (13.9)	85.3 (68.1-93.6)	85.3 (68.1-93.6)	2.320 (0.407-13.222)	0.3434
Poor (n = 29)	9 (31.0)	77.3 (56.3-89.1)	57.6 (31.8-76.7)	6.323 (1.064-37.563)	0.0425

*CRP* C-reactive protein, *eGFR* estimated glomerular filtration rate, *HR* hazard ratio, *IMDC* International Metastatic renal cell carcinoma Database Consortium, *NE* not estimable, *OS* overall survival

Best overall response was evaluable in 135 patients (Table 5). ORR in subgroups with favorable, intermediate (1 risk factor), intermediate (2 risk factors), and poor risk was 54.5% (95% CI, 36.4%-71.9%), 56.8% (95% CI, 41.0%-71.7%), 47.1% (95% CI, 29.8%-64.9%), and 54.2% (95% CI, 32.8%-74.4%), respectively, and disease control rate was 97.0% (95% CI, 84.2%-99.9%), 95.5% (95% CI, 84.5%-99.4%), 82.4% (95% CI, 65.5%-93.2%), and 75.0% (95% CI, 53.3%-90.2%), respectively. Complete response rate in subgroups with favorable, intermediate (1 risk factor), intermediate (2 risk factors), and poor risk was 12.1%, 6.8%, 8.8%, and 8.3%, respectively.

**Table 5:** Objective response in subgroups defined by IMDC risk classification^a^

	Favorable (n = 33)	Intermediate (1 risk factor) (n = 44)	Intermediate (2 risk factors) (n = 34)	Poor (n = 24)
Best overall response, n (%)				
Complete response	4 (12.1)	3 (6.8)	3 (8.8)	2 (8.3)
Partial response	14 (42.4)	22 (50.0)	13 (38.2)	11 (45.8)
Stable disease	14 (42.4)	17 (38.6)	12 (35.3)	5 (20.8)
Progressive disease	1 (3.0)	2 (4.5)	6 (17.6)	5 (20.8
Not evaluable	0	0	0	1 (4.2)
ORR, n (%) [95% CI]	18 (54.5) [36.4-71.9]	25 (56.8) [41.0-71.7]	16 (47.1) [29.8-64.9]	13 (54.2) [32.8-74.4]
DCR, n (%) [95% CI]	32 (97.0) [84.2-99.9]	42 (95.5) [84.5-99.4]	28 (82.4) [65.5-93.2]	18 (75.0) [53.3-90.2]

*DCR* disease control rate, *IMDC* International Metastatic renal cell carcinoma Database Consortium, *ORR* objective response rate

^a^Objective response was not reported in 15 patients in the overall population (6 patients with favorable risk, 2 patients with intermediate risk [1 risk factor], 2 patients with intermediate risk [2 risk factors], and 5 patients with poor risk

## Treatment for irAEs

Corticosteroid treatment at any dose was administered for irAEs in 22 patients (14.7%), with high-dose corticosteroid treatment administered in 11 (7.3%) (Table 6). In subgroups with favorable, intermediate (1 risk factor), intermediate (2 risk factors), and poor risk, respectively, corticosteroid treatment at any dose was administered in 8 (20.5%), 4 (8.7%), 5 (13.9%), and 5 (17.2%) patients, and high-dose corticosteroid treatment was administered in 2 (5.1%), 4 (8.7%), 3 (8.3%), and 2 (6.9%) patients. Durations of corticosteroid treatment are shown in Table 6.

**Table 6:** Use of corticosteroids for irAEs in subgroups defined by IMDC risk classification

	Favorable (n = 39)	Intermediate (1 risk factor) (n = 46)	Intermediate (2 risk factors) (n = 36)	Poor (n = 29)
Corticosteroids				
Patients, n (%)	8 (20.5)	4 (8.7)	5 (13.9)	5 (17.2)
Duration of treatment, median (range), months	1.9 (0.1-16.1)	7.7 (2.8-19.5)	0.8 (0.03-17.0)	2.6 (0.03-3.1)
High-dose corticosteroids^a^				
Patients, n (%)	2 (5.1)	4 (8.7)	3 (8.3)	2 (6.9)
Duration of treatment, median (range), months^b^	8.1 (0.1-16.1)	7.7 (2.8-19.5)	0.03 (0.03-0.8)	1.4 (0.03-2.8)

*IMDC* International Metastatic renal cell carcinoma Database Consortium, *irAE* immune-related adverse event

^a^High dose was defined as prednisolone-equivalent corticosteroid doses of ≥ 40 mg

^b^Duration period for ≥ 1 dose of high-dose corticosteroid

## Treatment discontinuations and subsequent treatment

At the end of the follow-up period, 75 patients (50.0%) were still receiving avelumab + axitinib, including 24 (61.5%) with favorable risk, 26 (56.5%) with intermediate (1 risk factor) risk, 16 (44.4%) with intermediate (2 risk factors) risk, and 9 (31.0%) with poor risk (Table 7). Common reasons for discontinuation were progressive disease in patients with intermediate (1 or 2 risk factors) or poor risk and AEs in patients with favorable risk. In subgroups with favorable, intermediate (1 risk factor), intermediate (2 risk factors), and poor risk, subsequent treatment was administered in 9 (23.1%), 18 (39.1%), 14 (38.9%), and 13 (44.8%) patients, respectively (Table 4). Cabozantinib was the most common subsequent treatment across subgroups, followed by nivolumab.

**Table 7:** Treatment discontinuation and subsequent treatments in subgroups defined by IMDC risk classification

	Favorable (n = 39)	Intermediate (1 risk factor) (n = 46)	Intermediate (2 risk factors) (n = 36)	Poor (n = 29)
Ongoing treatment, n (%)	24 (61.5)	26 (56.5)	16 (44.4)	9 (31.0)
Treatment discontinuation, n (%)	15 (38.5)	20 (43.5)	20 (55.6)	20 (69.0)
Reason for discontinuation, n (%)^a^				
Progression of disease	4 (10.3)	12 (26.1)	12 (33.3)	9 (31.0)
Adverse event	9 (23.1)	7 (15.2)	5 (13.9)	10 (34.5)
Other	2 (5.1)	2 (4.3)	4 (11.1)	4 (13.8)
Subsequent treatment, n (%)	9 (23.1)	18 (39.1)	14 (38.9)	13 (44.8)
Treatment regimen, n (%)				
Cabozantinib	5 (12.8)	13 (28.3)	12 (33.3)	8 (27.6)
Nivolumab	3 (7.7)	4 (8.7)	1 (2.8)	1 (3.4)
Nivolumab + cabozantinib	0	0	0	2 (6.9)
Axitinib	0	1 (2.2)	0	1 (3.4)
Pazopanib	0	0	1 (2.8)	1 (3.4)
Everolimus	1 (2.6)	0	0	0

*IMDC* International Metastatic renal cell carcinoma Database Consortium

^a^Patients with > 1 reason for discontinuation are included in all relevant rows

## Discussion

We report findings from a post hoc analysis of patient outcomes by IMDC risk classification using data from J-DART2, the largest real-world study to examine the long-term (observation period, ≥ 2 years) effectiveness of 1L avelumab + axitinib in patients with aRCC in Japan. Subgroup analyses by IMDC risk classification in J-DART2 confirm the effectiveness of 1L avelumab + axitinib observed in the JAVELIN Renal 101 clinical trial, including the subgroup analysis in patients enrolled in Japan [13, 15, 16].

Patient baseline characteristics in this study were generally well balanced across IMDC risk subgroups. However, the poor-risk subgroup had a higher proportion of patients with Eastern Cooperative Oncology Group performance status of ≥ 2, CRP level of ≥ 10 mg/L, and ≥ 2 metastatic lesions and a lower proportion of patients with prior nephrectomy. The age distribution across IMDC risk subgroups was also distinctive, with a high proportion of patients aged 65–74 years in all subgroups and a higher proportion of patients aged ≥ 75 years in intermediate-risk subgroups (1 or 2 risk factors). In subgroups with intermediate (1 risk factor), intermediate (2 risk factors), and poor risk, the most common IMDC factors were having < 1 year from time of diagnosis to systemic therapy and hemoglobin level < lower limit of normal.

The duration of avelumab + axitinib treatment in subgroups with favorable, intermediate (1 risk factor), and intermediate (2 risk factors) risk were similar and consistent with that reported in the overall population (12.6, 12.0, and 10.5 vs 10.7 months) [19]; the duration of treatment was shorter in the poor-risk subgroup (8.0 months). Although OS was not reached in any subgroup, the 12-month OS rate in subgroups with favorable, intermediate (1 risk factor), intermediate (2 risk factors), and poor risk were 100%, 95.3%, 85.3%, and 77.3%, respectively, which was comparable to the 12-month OS rate in the overall population from JAVELIN Renal 101 (86.2%) and subgroups by the number of IMDC risk factors (95.7% [favorable- 0 risk factor], 89.5% [intermediate-1 risk factor], 85.4% (intermediate- 2 risk factors), 65.9% (poor- 3 risk factors), and 59.9% (poor- 4–6 risk factors) [20, 22]. The 12-month OS rates were also comparable to results from real-world postmarketing surveillance in Japan; in subgroups with favorable, intermediate (1 risk factor), intermediate (2 risk factors), and poor risk, 12-month OS rates were 97.7%, 92.9%, 79.5%, and 54.8%, respectively [17]. In addition, J-DART2 demonstrated the long-term clinical outcomes of avelumab + axitinib in subgroups with favorable, intermediate (1 risk factor), intermediate (2 risk factors), and poor risk, with 2-year survival rates of 95.2%, 91.3%, 85.3%, and 57.6%, respectively. In exploratory multivariable analyses, no significant OS differences were observed between the favorable and the intermediate IMDC risk subgroups. These findings suggest that avelumab + axitinib is associated with clinically meaningful benefits across IMDC risk subgroups, including patients with favorable, intermediate (1-risk factor), and intermediate (2-risk factors) IMDC risk classification.

In addition, multivariable analyses showed a significant difference in the HR for OS in patients with eGFR of < 30 vs ≥ 60 mL/min, which is likely due to differences in renal function. Data regarding the association of kidney function with outcomes of ICI-based combination therapy for aRCC are limited. A previous study suggested that the efficacy of ICI + TKI combination therapies is not associated with differences in patients with chronic kidney disease, but that the safety profile may differ based on the baseline renal function [23]. However, more data are needed to fully understand the effects of renal function on the efficacy of ICI + TKI combination therapies.

ORR and disease control rate in J-DART2 were consistent across IMDC risk subgroups. However, median PFS was longer in the favorable-risk subgroup vs the subgroups with intermediate (1 risk factor), intermediate (2 risk factors), or poor risk (31.0 vs 15.3, 16.4, or 8.1 months, respectively), but values were comparable to those reported in subgroup analyses from the JAVELIN Renal 101 trial [13, 15]. In exploratory multivariable analyses, significant differences were observed in the HRs for long-term PFS and OS in the poor- vs favorable-risk subgroup, which may be due to differences in patient baseline and disease characteristics.

After long-term follow-up in J-DART2, the proportion of patients who received high-dose corticosteroid treatment for irAEs with avelumab + axitinib was generally consistent across IMDC risk subgroups (5.1%-8.7%) and was comparable to those in the overall population of JAVELIN Renal 101 (14.5%) and the subgroup analysis in Japan (9.1%) as well as the real-world J-DART study (6.3%) [13, 16, 18]. The duration of corticosteroid and high-dose corticosteroid treatment for irAE was also consistent across all IMDC risk subgroups. These data suggest that irAEs are appropriately managed across IMDC risk subgroups in clinical practice in Japan.

Treatment guidelines recommend several ICI + TKI combinations across IMDC risk groups based on results from phase 3 trials [1–3, 24–26]. Given the prognostic utility of IMDC risk and the heterogeneity of patient populations in clinical practice, real-world data on treatment outcomes by IMDC risk classification are needed to help guide more individualized treatment strategies. In subgroup analyses from J-DART2, 1L avelumab + axitinib was associated with clinically meaningful benefits across IMDC risk subgroups, and clinical outcomes were consistent with those of previous studies, including subgroup analyses by IMDC risk from the JAVELIN Renal 101 clinical trial and outcomes from the real-world J-DART study [13, 15, 18].

Our study had some limitations. As a retrospective study, only existing data reported in patient records were available for analyses, and missing data may have affected the accuracy of estimations. Different methodologies for evaluating disease response across study sites may have led to variations in estimated values. High-volume centers were preferentially selected for this study, which could potentially result in site-selection and outcome-reporting biases. Therefore, the study results may not accurately reflect outcomes in all patients with aRCC in clinical practice in Japan. In addition, J-DART2 did not collect data on AEs to avoid overlap with a postmarketing surveillance study that has analyzed AE data for avelumab + axitinib in clinical practice in Japan [17].

## Conclusion

Subgroup analyses by IMDC risk classification from the J-DART2 study provide real-world data on patient characteristics and the long-term effectiveness of 1L avelumab + axitinib in patients with aRCC in clinical practice in Japan. Avelumab + axitinib was associated with clinically meaningful efficacy benefits across IMDC risk subgroups, and outcomes were generally consistent with those reported previously. High-dose corticosteroid use to manage irAEs was low. Findings from J-DART2 support the continued use of avelumab + axitinib as a 1L treatment for patients with aRCC, regardless of IMDC risk classification.

## Supplementary Information

Below is the link to the electronic supplementary material.

Supplementary file1 (DOCX 23 KB)

## Data Availability

Any requests for data by qualified scientific and medical researchers for legitimate research purposes will be subject to Merck’s Data Sharing Policy. All requests should be submitted in writing to Merck’s data sharing portal (https://www.merckgroup.com/en/research/our-approach-to-research-and-development/healthcare/clinical-trials/commitment-responsible-data-sharing.html). When Merck has a co-research, co-development, or co-marketing or co-promotion agreement, or when the product has been out-licensed, the responsibility for disclosure might be dependent on the agreement between parties. Under these circumstances, Merck will endeavor to gain agreement to share data in response to requests.
