# Supplementary material for: Real-world outcomes with avelumab + axitinib in patients with advanced renal cell carcinoma in Japan: subgroup analyses from the J-DART2 study by International Metastatic Renal Cell Carcinoma Database Consortium risk classification
Source: Int J Clin Oncol. 2025 Feb 11;30(4):749–60. doi: 10.1007/s10147-024-02655-4 (PMC11946980; doi:10.1007/s10147-024-02655-4)
Supplement: Supplementary file 1 — Supplementary file1 (DOCX 23 KB) [file 10147_2024_2655_MOESM1_ESM.docx]

**Original Article**

**Real-world outcomes with avelumab + axitinib in patients with advanced renal cell carcinoma in Japan: subgroup analyses from the J-DART2 study by International Metastatic Renal Cell Carcinoma Database Consortium risk classification**

**Authors:**

| **Name** | **Affiliation address** | **Email address/phone number** |
| --- | --- | --- |
| Junya Furukawa | Department of Urology, Kobe University Graduate School of Medicine, 7-5-1 Kusunoki-cho, Chuo-ku, Kobe, 650-0017, Japan | [jfuru@med.kobe-u.ac.jp](mailto:jfuru@med.kobe-u.ac.jp)  +81-78-382-5111 |
| Taigo Kato | Department of Urology, Osaka University Graduate School of Medicine, 2-2 Yamadaoka,  Suita, Oska, 565-0871, Japan | [kato@uro.med.osaka-u.ac.jp](mailto:kato@uro.med.osaka-u.ac.jp)  +81-66-879-5111 |
| Toshinari Yamasaki | Department of Urology, Kobe City Medical Center General Hospital, 2-1-1 Minatojima-Minamimachi, Chuo-ku, Kobe, 650-0047, Japan | [toshinari_yamasaki@kcho.jp](mailto:toshinari_yamasaki@kcho.jp) +81-78-302-4321 |
| Keisuke Monji | Department of Urology, Graduate School of Medical Sciences, Kyushu University, 3-1-1 Maidashi, Higashi-ku, Fukuoka, 812-8582, Japan | monji_jr@hotmail.com +81-92-642-5615 |
| Toshiaki Tanaka | Department of Urology, Sapporo Medical University, South-1, West-16, Chuo-ku, Sapporo, Hokkaido 060-8543, Japan | [ttoshizappa@gmail.com](mailto:ttoshizappa@gmail.com) +81-11-611-2111 |
| Norihiko Tsuchiya | Department of Urology, Faculty of Medicine, Yamagata University, 2-2-2, Iida-Nishi, Yamagata 990-9585, Japan | [ntsuchiya@med.id.yamagata-u.ac.jp](mailto:ntsuchiya@med.id.yamagata-u.ac.jp) +81-23-628-5368 |
| Tomoaki Miyagawa | Department of Urology, Jichi Medical University Saitama Medical Center, 1-847, Amanuma-cho, Omiya-ku, Saitama-shi, 330-8503, Saitama, Japan | [sh2-miya@jichi.ac.jp](mailto:sh2-miya@jichi.ac.jp) +81-48-647-2111 |
| Hiroshi Yaegashi | Department of Integrative Cancer Therapy and Urology, Kanazawa University Graduate School of Medical Sciences, 13‐1 Takaramachi, Kanazawa City, Ishikawa, Japan | [hyae2002jp@yahoo.co.jp](mailto:hyae2002jp@yahoo.co.jp) +81-76-265-2393 |
| Tomoyasu Sano | Department of Urology, Nagoya University Graduate School of Medicine, 65 Tsurumai-cho, Showa-ku, Nagoya 466-8550, Japan | [t-sano@med.nagoya-u.ac.jp](mailto:t-sano@med.nagoya-u.ac.jp)  +81-52-741-2111 |
| Takashi Karashima | Department of Urology, Kochi Medical School, Kohasu, Nankoku, Kochi, 783-8505, Japan | [karasima@kochi-u.ac.jp](mailto:karasima@kochi-u.ac.jp) +81-88-880-2402 |
| Kazutoshi Fujita | Department of Urology, Kindai University Faculty of Medicine, 377-2 Ohno-Higashi, Osakasayama City, Osaka 589-8511, Japan | [kfujita@med.kindai.ac.jp](mailto:kfujita@med.kindai.ac.jp) +81-72-366-0221 |
| Jun-ichi Hori | Department of Renal and Urologic Surgery, Asahikawa Medical University, 2-1-1-1 Midorigaoka Higashi, Asahikawa, Hokkaido 078-8510, Japan | [urohori@asahikawa-med.ac.jp](mailto:urohori@asahikawa-med.ac.jp) +81-166-65-2111 |
| Takayuki Ito | Medical Department, Merck Biopharma Co., Ltd., Tokyo, Japan, an affiliate of Merck KGaA, 1-8-1 Shimomeguro, Meguro-ku, Tokyo 153-8926, Japan | [takayuki.ito@merckgroup.com](mailto:takayuki.ito@merckgroup.com)  +81-3-6756-0800 |
| Masahiro Kajita | Medical Department, Merck Biopharma Co., Ltd., Tokyo, Japan, an affiliate of Merck KGaA, 1-8-1 Shimomeguro, Meguro-ku, Tokyo 153-8926, Japan | [masahiro.kajita@merckgroup.com](mailto:masahiro.kajita@merckgroup.com)  +81-3-6756-0800 |
| Yoshihiko Tomita | Departments of Urology and Molecular Oncology, Niigata University Graduate School of Medical and Dental Sciences, 1-757 Asahimachi Street, Niigata City, Chuo Ward, Japan 951-8510 | [ytomita@med.niigata-u.ac.jp](mailto:ytomita@med.niigata-u.ac.jp)  +81-25-227-2289 |
| Nobuo Shinohara | Department of Urology, Graduate School of Medicine, Hokkaido University, Kita15, Nishi7, Kita-Ku, Sapporo Hokkaido 060-8638, Japan | [nozomis@mbj.nifty.com](mailto:nozomis@mbj.nifty.com)  +81-11-706-5966 |
| Masatoshi Eto | Department of Urology, Graduate School of Medical Sciences, Kyushu University, 3-1-1 Maidashi, Higashi-ku, Fukuoka, 812-8582, Japan | [eto.masatoshi.717@m.kyushu-u.ac.jp](mailto:eto.masatoshi.717@m.kyushu-u.ac.jp)  +81-92-642-5615 |
| Mototsugu Oya | Department of Urology, Keio University School of Medicine, 35 Shinanomachi, Shinjuku-ku, Tokyo 160-8582, Japan | [moto-oya@z3.keio.jp](mailto:moto-oya@z3.keio.jp)  +81-3-3353-1211 |
| Hirotsugu Uemura | Department of Urology, Kindai University Faculty of Medicine, 377-2 Ohno-Higashi, Osakasayama City, Osaka 589-8511, Japan | [huemura@med.kindai.ac.jp](mailto:huemura@med.kindai.ac.jp)  +81-72-366-0221 |

**Target journal:** [*Int J Clin Oncol*](https://link.springer.com/journal/10147/submission-guidelines#Instructions%20for%20authors_Manuscript%20preparation) (Original Article)

**Corresponding author:** Hirotsugu Uemura, Department of Urology, Kindai University Faculty of Medicine, 377-2 Ohno-Higashi, Osakasayama City, Osaka 589-8511, Japan

Email: [huemura@med.kindai.ac.jp](mailto:huemura@med.kindai.ac.jp)

Tel: +81-72-366-0221

ORCiD: 0000-0002-3665-9523

**Supplementary Table** Distribution of risk factors in subgroups with intermediate (1 or 2 risk factors) or poor IMDC risk classification. **C**, calcium level > upper limit of normal; **H**, hemoglobin level < lower limit of normal; **K**, Karnofsky performance status <80%; **L**, <1 year from time of diagnosis to systemic therapy; **N**, neutrophil count > upper limit of normal; **P**, platelet count > upper limit of normal

| **Risk factor** | **n (%)** |
| --- | --- |
| **Intermediate (1 risk factor)** | **n=46** |
| L | 24 (52.2) |
| H | 17 (37.0) |
| N | 5 (10.9) |
| **Intermediate (2 risk factors)** | **n=36** |
| LH | 23 (63.9) |
| LK | 2 (5.6) |
| LC | 2 (5.6) |
| LN | 1 (2.8) |
| LP | 1 (2.8) |
| KH | 1 (2.8) |
| HN | 3 (8.3) |
| HP | 2 (5.6) |
| CP | 1 (2.8) |
| **Poor** | **n=29** |
| LHN | 5 (17.2) |
| LHC | 3 (10.3) |
| LHP | 3 (10.3) |
| LHK | 2 (6.9) |
| LCP | 1 (3.4) |
| LNP | 1 (3.4) |
| KHC | 1 (3.4) |
| KHN | 1 (3.4) |
| HCP | 2 (6.9) |
| LHNP | 3 (10.3) |
| LHCP | 2 (6.9) |
| LKHC | 2 (6.9) |
| KHCN | 1 (3.4) |
| HCNP | 1 (3.4) |
| LKCNP | 1 (3.4) |
